# Exploiting genomic synteny in *Felidae*: cross-species genome alignments and SNV discovery can aid conservation management

**DOI:** 10.1186/s12864-021-07899-2

**Published:** 2021-08-06

**Authors:** Georgina Samaha, Claire M. Wade, Hamutal Mazrier, Catherine E. Grueber, Bianca Haase

**Affiliations:** 1 Sydney School of Veterinary Science, Faculty of Science, The University of Sydney, Sydney, NSW Australia; 2 School of Life and Environmental Sciences, The University of Sydney, Sydney, NSW Australia

**Keywords:** Genomics, Conservation, Felids, Cheetah, Tiger, Snow leopard, Cat, Cross-species, SNV, WGS

## Abstract

**Background:**

While recent advances in genomics has enabled vast improvements in the quantification of genome-wide diversity and the identification of adaptive and deleterious alleles in model species, wildlife and non-model species have largely not reaped the same benefits. This has been attributed to the resources and infrastructure required to develop essential genomic datasets such as reference genomes. In the absence of a high-quality reference genome, cross-species alignments can provide reliable, cost-effective methods for single nucleotide variant (SNV) discovery. Here, we demonstrated the utility of cross-species genome alignment methods in gaining insights into population structure and functional genomic features in cheetah (*Acinonyx jubatas*), snow leopard (*Panthera uncia*) and Sumatran tiger (*Panthera tigris sumatrae*), relative to the domestic cat (*Felis catus*).

**Results:**

Alignment of big cats to the domestic cat reference assembly yielded nearly complete sequence coverage of the reference genome. From this, 38,839,061 variants in cheetah, 15,504,143 in snow leopard and 13,414,953 in Sumatran tiger were discovered and annotated. This method was able to delineate population structure but limited in its ability to adequately detect rare variants. Enrichment analysis of fixed and species-specific SNVs revealed insights into adaptive traits, evolutionary history and the pathogenesis of heritable diseases.

**Conclusions:**

The high degree of synteny among felid genomes enabled the successful application of the domestic cat reference in high-quality SNV detection. The datasets presented here provide a useful resource for future studies into population dynamics, evolutionary history and genetic and disease management of big cats. This cross-species method of variant discovery provides genomic context for identifying annotated gene regions essential to understanding adaptive and deleterious variants that can improve conservation outcomes.

**Supplementary Information:**

The online version contains supplementary material available at 10.1186/s12864-021-07899-2.

## Background

As natural habitats and ecosystems are increasingly impacted by anthropogenic events, a growing number of species require some form of ex situ management to prevent extinction. Big cat species are amongst the most vulnerable, having experienced dramatic population declines as result of illegal poaching, trade and habitat destruction. All 38 wild felid species have a negative global population trend [1]. Despite these challenges, wild felids are revered as cultural symbols and are important flagship species for engendering public interest in conservation programmes. As keystone species, strategies for the protection of large felids also benefit their ecosystems [2]. Allelic variation is essential to preserving species’ genetic integrity and maintaining functioning ecosystems, as genetically diverse populations tend to have higher fitness and adaptive capacity [3]. Given their conservation status, many big cats are the subjects of Species Survival Plans (SSP) [4, 5]; internationally coordinated programmes that manage ex situ breeding and aim for healthy, self-sustaining and genetically diverse populations. Retaining genetic diversity in a closed, captive population is challenging and expensive [6, 7]. Reductions in genetic diversity due to inbreeding, genetic drift and selection can lead to an accumulation of deleterious mutations in a captive-bred population, threatening their long-term viability [8, 9].

With genomic resources becoming increasingly accessible for a wide diversity of species, single nucleotide variant (SNV)-based genetic analyses offer conservationists higher resolution for measuring diversity and addressing conservation questions than previously [10]. In model species, SNVs are the genetic marker of choice for the advancement of functional, quantitative and evolutionary research [11–13]. Their high frequency across coding and non-coding regions, low typing error rates and ease of comparability across datasets, make them a favourable alternative or complement to low density markers such as microsatellites, allozymes and mtDNA [14]. Genome-wide SNV discovery methods have enabled the transition from estimates of inbreeding using pedigree data and microsatellite markers, to the direct quantification of inbreeding via genome-wide scans of individual homozygosity [15–17]. The use of genome-wide SNV data in wild cats has illuminated patterns of genetic variation relating to population history, physiological adaptation and speciation and the extent of genomic synteny among *Felidae* [18–22]. However, there remains a notable gap between these studies and the integration of genomics into the conservation management of these species. Recent reviews have highlighted several barriers to the widespread uptake of genomic data in conservation, including: high costs associated with sequencing and sampling, lack of computational infrastructure, need for specialist bioinformatic expertise, and the absence of genomic resources (i.e. high-quality reference data) for non-model species [23, 24]. Here, ‘non-model species’ refers to those with limited genomic resources, specifically reference genomes.

A shortage of high-quality reference genomes is often cited as a barrier to SNV discovery and genotyping in non-model species [24–26]. Reference-guided SNV discovery is more computationally efficient than methods that do not use a reference, offering higher accuracy at lower sequencing depths, as well as the ability to physically map and determine linkage disequilibrium between SNVs. The genomic context provided by a reference genome allows for identifying annotated gene regions essential to understanding any potential adaptive or deleterious variant consequences. Compared with many other carnivorous genera, felid genomics has been extensively studied: a number of domestic cat (*Felis catus*) reference assemblies have been developed since 2007 [27], followed by draft assemblies of tiger (*Panthera tigris)* [20], cheetah *(Acinonyx jubatus)* [18], leopard (*Panthera pardus)* [28], jaguar (*Panthera onca)* [21] and lion (*Panthera leo*) [29], among others. Compared with its wild counterparts, the most recent domestic cat reference assembly (felCat9) is a chromosome-level build that offers fewer gaps at a higher sequencing depth. Generating these reference genomes required considerable financial, computational and bioinformatic resources, rarely available to conservation managers. High-quality genome assemblies are differentiated from draft-quality assemblies by their lower error rates, fewer gaps (e.g. chromosome-level assembly), and high-quality annotations. Recently, a correction of the tiger draft assembly was published [30], highlighting the potential for inadequately validated draft assemblies to bias the outcomes of genomic observations and biological conclusions.

Cross-species reference-guided SNV calling and genotyping methods [31–34], reduced-representation libraries [19, 35, 36] and pooled sequencing (poolseq) [37, 38] have successfully been used to study genomics in non-model species, circumventing the costs, computational resources and extensive sampling required to develop a high quality reference genome. A high degree of genomic synteny has been observed across felid species using cytological and genomic approaches [18, 20, 29, 39–41]. Here, we took advantage of this conserved genomic architecture among felids and performed cross-species whole genome sequence (WGS) alignment and SNV discovery using the domestic cat reference genome assembly and WGS data of three big cat species; Sumatran tiger (*Panthera tigris sumatrae*), snow leopard (*Panthera uncia*) and cheetah. We compare the utility of non-barcoded pooled versus individual WGS data for cross-species alignment and SNV discovery. We present an annotated catalogue of high-quality variants from seventeen whole genome sequences comprising twenty-six individuals across the three species. We show that reference genomes from distantly related species can be successfully used for SNV discovery to inform conservation management. Gene enrichment and gene diversity analyses provided a proof of principle of the effectiveness of cross-species application of the domestic cat reference genome in variant calling in big cat species.

## Results

### Cross-species WGS alignment and variant calling using the felCat9 reference assembly

Genomic DNA of four Sumatran tigers, four cheetahs and four snow leopards was pooled by species in equimolar ratios and sequenced. Whole genome sequences for seven Sumatran tigers, six cheetahs and one snow leopard were downloaded from sequence read archive (SRA) [42]. Individual and pooled samples were aligned to the felCat9 [43] reference assembly. The number of reads in each species pool was 830 M for cheetah, 960.7 M for snow leopard and 896.7 M for Sumatran tiger. Overall sequencing performance is summarised in Table S1 and results for alignments and variant calling for all individuals and pools are presented in Table 1. An average of 170 M cheetah reads, 627 M snow leopard reads, and 251 M Sumatran tiger reads were mapped to the Felis_catus_9.0 (felCat9; GCA_000181335.4) reference assembly. On average, 94 % of cheetah, 93 % of snow leopard and 95 % of Sumatran tiger reads were properly paired and mapped to felCat9 chromosomes (Figure S1). The proportion of successfully paired and mapped reads was lower for Sumatran tiger and cheetah pools compared with their individually sequenced counterparts. The read depth for all individual samples ranged between 5.56x and 35.91x. Genome coverage was highest over a greater portion of bases for all species pools compared with their SRA counterparts (Figure S2). Read coverage for species pools ranged from 25.78x to 50.14x and alignment of species pools to the felCat9 reference assembly resulted in greater than 90% coverage of the reference at a minimum depth of 20x compared with a depth of ~ 5x, ~7x and 10x in cheetah, Sumatran tiger and snow leopard individual samples, respectively.

**Table 1:** Summary of alignment of big cat individuals and pools to the domestic cat reference assembly

Species	Sample ID	Reads mapped (%)	Read depth	Quality filtered SNVs	TsTv ratio	Nucleotide diversity (π)
Cheetah	CHEETAH_NAM1	97.74	5.56	27,854,391	1.82	0.00596
	CHEETAH_NAM2	97.44	7.15	31,254,807	1.83	0.00804
	CHEETAH_NAM3	97.45	8.55	32,936,565	1.85	0.00940
	CHEETAH_TZA1	96.94	7.15	29,981,645	1.81	0.00765
	CHEETAH_TZA2	97.41	6.28	27,435,836	1.80	0.00637
	CHEETAH_TZA3	97.35	7.33	31,533,437	1.85	0.00801
	pool (*N *= 4)	79.23	43.59	38,246,085	2.12	0.04701
Sumatran tiger	SUM_IDN1	95.20	12.41	2,245,646	1.70	0.00243
	SUM_IDN2	96.94	13.43	2,984,324	1.67	0.00237
	SUM_IDN3	93.87	11.75	2,213,024	1.79	0.00213
	SUM_IDN4	95.43	11.93	2,626,994	1.60	0.00243
	SUM_IDN5	94.98	12.51	2,442,662	1.78	0.00194
	SUM_USA1	94.47	11.46	2,205,499	1.73	0.00203
	SUM_USA2	96.58	12.42	2,361,962	1.68	0.00243
	pool (*N* = 4)	88.84	25.78	18,859,425	2.10	0.00740
Snow leopard	SNOW	91.30	43.59	10,634,946	1.73	0.00246
	pool (*N* = 4)	95.58	50.14	9,340,412	1.83	0.00281

To compare the utility of non-barcoded pooled versus individual WGS, variant calling was performed in diploid mode for all samples. Across Sumatran tiger and cheetah samples, fewer biallelic variants were called for individuals than for their pooled counterparts, while the snow leopard pool and individual samples showed approximately equal numbers of variants (Table 1). Individual nucleotide diversity (π) is reported under the assumption that both parental sets of chromosomes have been sequenced to equivalent coverage depths. This statistic was similar among pools and individuals for Sumatran tigers and snow leopards, however the cheetah pool displayed markedly higher π compared to all sequenced individuals and compared to pools for the other two species. Despite having the lowest average read coverage, cheetahs had the highest proportion of reads mapped and high-quality SNVs called against the felCat9 reference assembly (Table 1). Cheetahs had a higher density of SNVs per kilobase across all felCat9 chromosomes than Sumatran tigers and snow leopards. The cheetah and Sumatran tiger pools had a significantly higher density of SNVs across all chromosomes compared with individuals of the respective species. In total, 13,414,953, 15,504,143 and 38,839,061 biallelic SNVs passed quality filtering in Sumatran tiger, snow leopard and cheetah, respectively. Of these, 10,472,528 in Sumatran tigers, 9,124,699 in snow leopards and 26,430,702 in cheetahs were transitions (Ts) and 5,030,622 in Sumatran tigers, 4,285,891 in snow leopards and 12,258,571 in cheetahs were transversions (Tv). Ts/Tv ratios for pooled samples were higher than individual samples (Table 1). Sumatran tiger individuals had a mean Ts/Tv ratio of 1.7 (σ = 0.06) while the Sumatran tiger pool reported a Ts/Tv ratio of 2.09. Cheetah individuals similarly reported a lower mean Ts/Tv ratio of 1.8 (σ = 0.02) compared with their pooled counterparts, which had a Ts/Tv ratio of 2.1, potentially indicating a higher rate of false variants in the individual sample variant sets. Ts/Tv ratios for species variant sets ranged from 2.08 to 2.15.

### Population structure and demographic statistics

**Fig. 1:**
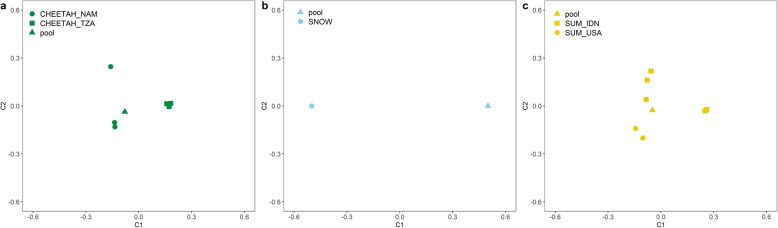
Distinct clustering of samples within species groups based on multi-dimensional scaling (MDS). **a** Cheetah individuals are labelled as either Namibian (CHEETAH_NAM) or Tanzanian (CHEETAH_TZA) based on geographic provenance, **b** Snow leopard pool and individual samples and **c** Sumatran tiger individuals were also labelled as either American (SUM_USA) or Indonesian (SUM_IND) based on geographic provenance

Population structure and demographic statistics were calculated using minor allele frequency (MAF) filtered datasets for all species. Multi-dimensional scaling (MDS) was used to partition total genomic variation among individuals in each species group (Fig. 1). There was clear differentiation between individuals and pools in all three species, and among individuals of different geographical provenance. The first component (C1) corresponded to the axis of differentiation among Tanzanian and Namibian cheetahs, with the pool distinctly clustering with Namibian cheetahs. Among Sumatran tigers, the second component (C2) accounted for differentiation between Indonesian (IDN) and American (USA), with two distinct clusters of Indonesian individuals separated by C1.

Estimates of genetic variability and pairwise similarity were measured in Sumatran tiger and cheetah individuals (only one individual sample was available for snow leopard) (Table 2). Co-ancestry coefficients (ϴ) were calculated as the probability of finding identical alleles when randomly sampling one allele from each heterozygous individual and suggested unrelated kinship among all Sumatran tiger individuals. Expected (He) and observed heterozygosity (Ho) were calculated to measure genetic diversity of each population. Ho was lower than He across all samples, indicating a deviation from Hardy-Weinberg equilibrium (HWE) and possible inbreeding (non-random mating). Mean individual inbreeding coefficients (F) among cheetahs was 0.346 ± 0.062 (range:0.259–0.419) and 0.585 ± 0.033 (range:0.233–0.273) among Sumatran tigers. These results may reflect close relationships among samples within each species, however this cannot be confirmed as data regarding their pedigree was not made available. The proportion of pairwise identity by state (IBS) allelic similarity among cheetahs ranged from 0.472 to 0.394 (µ = 0.437 ± 0.028). Mean IBS among Namibian cheetahs was 0.412 and 0.398 among Tanzanian cheetahs. Among Sumatran tigers IBS ranged from 0.392 to 0.521 (µ = 0.5 ± 0.026).

**Table 2:** Heterozygosity, pairwise relatedness and similarity coefficients of each species group was calculated using individual data only (i.e. pools excluded). Heterozygous SNV rate was calculated as the ratio of heterozygous SNVs over the felCat9 genome assembly length. Snow leopard values correspond to the single individual sample ‘SNOW’

Within species	IBS (mean ± SD)	Coancestry coefficient (ϴ) (mean ± SD)	Expected heterozygosity (mean ± SD)	Observed heterozygosity (mean ± SD)	Heterozygous SNV rate	Inbreeding coefficient (F) (mean ± SD)	FMin	FMax
Snow leopard (*N* = 1)	0.5	-	0.49	0.38	0.004	0.233	-	-
Sumatran tiger (*N* = 7)	0.43 ± 0.16	1.11 ± 0.44	0.429 ± 0.0002	0.178 ± 0.014	0.001 ± 0.0001	0.585 ± 0.033	0.553	0.645
Cheetah (*N* = 6)	0.36 ± 0.14	-0.42 ± 0.31	0.392 ± 0.0002	0.245 ± 0.025	0.012 ± 0.0008	0.346 ± 0.062	0.259	0.419

### Functional annotation of genomic variants

**Fig. 2:**
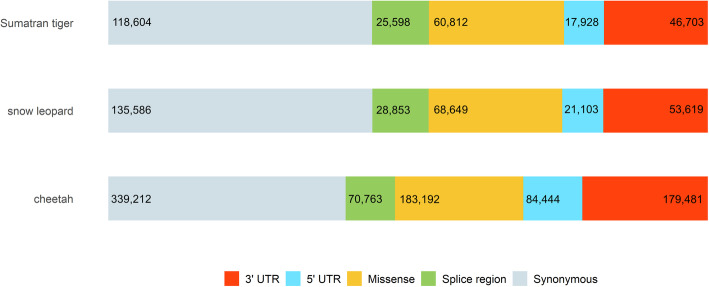
Distribution of major transcript-associated SNV annotation categories among all three big cat species was proportional among each species

Variant datasets were annotated using Variant Effect Predictor (VEP) in order to identify coding variants of potential functional significance. VEP assigned 29,059,874, 25,194,581 and 73,915,269 functional classes to SNVs in snow leopards, Sumatran tigers and cheetahs, respectively, based on the felCat9 reference assembly annotation. The number of functional classes defined by VEP is higher than the total number of SNVs, because some sites have multiple annotations. The quantity of quality-filtered, fixed and MAF-filtered variants annotated by VEP varied among species, however the number of transcripts and genes overlapped were consistent across all species (Table 3) and functional annotation of transcript-associated variants was similar across all three species, with over 60 % of coding variants labelled as synonymous for all species (Fig. 2). A summary of functional annotation of MAF-filtered and fixed SNVs is provided for each species dataset (Table S2).

**Table 3:** Summary of SNVs called and annotated for each species. Total number of samples corresponds to the number of individual WGS and individuals included in each pool (*N*=4). Fixed SNVs refers to variants calling the alternative allele across all samples in each species relative to the domestic cat

	Cheetah	Snow leopard	Sumatran tiger
**Quality filtered SNV**			
**Total**	38,839,061	15,504,143	13,414,953
**Coding variants**	449,996	144,156	126,127
**Transcripts overlapped**	53,786	53,707	53,640
**Genes overlapped**	29,043	28,978	28,930
**Fixed SNV**			
**Total**	1,737,447	13,882,181	3,755,816
**Coding variants**	9,910	122,446	37,539
**MAF filtered SNV**			
**Total**	2,671,858	55,604	409,014
**MAF threshold**^**a**^	0.125	0.25	0.143
**Missense**	17,333	623	2,737
**Deleterious**^**b**^	4,734	177	772

^a^MAF thresholds were used to exclude SNV that did not appear in at least one individual in a homozygous state or at least two individuals in a heterozygous state

^b^SNV annotated as ‘deleterious’ by VEP tool had a SIFT score 0-0.05

Cheetah MAF-filtered variants were enriched for over 90 terms that were reduced to best representative terms: macromolecular localisation (GO:0033036; P_adj_=5.67 × 10^− 9^), regulation of biological quality (GO:0065008; P_adj_=7.36 × 10^− 8^), cytoskeleton organisation (GO:0007010; P_adj_=5.09 × 10^− 9^), developmental process (GO:0032502; P_adj_=7.91 × 10^− 17^) and cytoskeleton organisation (GO:0005856, P_adj_=9.20 × 10^− 12^) (Table S3a). In Sumatran tigers, variants passing the MAF-filter were enriched for protein binding (GO:0005515; P_adj_=3.54 × 10^− 4^), cilium (GO:0005929; P_adj_=1.53 × 10^− 4^), and collagen-containing extracellular matrix (GO:0062023; P_adj_=3.53 × 10^− 4^) (Table S3b). The snow leopard MAF-filtered dataset was not found to be functionally enriched. Calling variants from one snow leopard individual and a pool called in diploid mode has likely confounded the number of fixed and MAF-filtered variants in snow leopards. In snow leopard, a lack of significant functional enrichment among genes containing MAF-filtered variants may be attributed to small sample size.

### Genomic signatures of adaptation among big cats

Signatures of selection within each species were tested using nucleotide and gene diversity of synonymous and non-synonymous SNVs. Most genes had a pairwise nucleotide diversity (π_N_/π_S_) < 1 while 54 genes in cheetahs, six genes in Sumatran tigers and one gene in snow leopards revealed signs of positive selection (Table S4). All genes displaying signatures of positive selection in tiger and snow leopard were involved in olfaction. In cheetahs, genes showing signs of selection were involved in olfaction (*LOC101084218, LOC101085032, LOC101086178, LOC101095034* and *LOC101101377*) and immune responses (*TLR3, SYTL2, OAS3* and *RAB44*). Genes under positive selection also included dynein axonemal heavy chain genes *DNAH2* and *DNAH6*, involved in flagellum-dependent cell motility (GO:0001539) and *SGCG* and *XIRP1*, expressed exclusively in skeletal muscle.

SNVs fixed in each species group were collected and annotated to identify genes potentially involved in species-specific phenotypic signatures of adaptation. This also served to highlight phenotypic differences between the domestic cat and big cat species. In cheetahs, genes harbouring homozygous SNVs related to KEGG pathways were: *HEXB, HARS1, PPP2CA, TGDS*, *ALG11* and *PDE4D*. Fixed missense alleles in cheetahs also occurred in *ACTN3*, previously associated with athletic performance in humans and horses [44, 45]. When reduced to the most representative subset of GOterms by semantic similarity, a total of 144 representative GO terms were analysed for fixed SNVs in Sumatran tigers and 95 in snow leopards (Table S5a,b). In Sumatran tigers, genes harbouring fixed variants were enriched for metabolic pathways (KEGG:01100; P_adj_=1.94 × 10^− 17^). Fixed non-synonymous alleles shared by snow leopards and Sumatran tigers were enriched for growth (GO:0040007), locomotion (GO:0040011), and developmental process (GO:0032502). These included missense variants in genes previously associated with body size: *LCORL* that were unique to *Panthera* species (Table S6). Pathway enrichment analysis was performed to gain mechanistic insight into genes containing fixed variants. Fixed non-synonymous variants in 418 genes common to all three big cat species were annotated for cadherin signalling (P_adj_=1.51 × 10^− 5^) and Wnt signalling (P_adj_=0.001) pathways. Genes in the protocadherin family displayed fixed allelic differences between all big cats and the domestic cat genome (Table S7).

### Genetic insights into heritable conditions affecting big cats

To identify genes potentially underlying conditions of clinical importance in each species, a non-redundant list of genes categorised by GO terms relevant to known heritable conditions, immune and reproductive function was collected for each species. Genes containing deleterious variants (SIFT score 0-0.05) were collected from MAF-filtered datasets and observed in 201 genes annotated for relevant GO terms in cheetah, in six genes in snow leopards and in 44 genes in Sumatran tigers (Table S8). In cheetahs, these genes were annotated for spermatogenesis (GO:0007283), cilium assembly (GO:0060271), sperm flagellum (GO:0036126), B cell mediated immunity (GO:0019724) and embryo development (GO:0009790) (Figure S3a). In cheetahs, genes housing variants previously associated with known disease relating to ciliary dysfunction included *PCDH15*, *HOMER2*, *SPEF2, NAGLU*, *PHGDH*, *ATR* and *ABL1*. In Sumatran tigers, genes containing deleterious variants were restricted to terms relating to cilium structure and assembly (GO:0060271, GO:0032420) (Figure S3b) and *ATR* was also the only gene included in Sumatran tiger phenotype-associated genes. Snow leopard genes containing deleterious variants were: *ADAM29, ADCY10, CCNYL1, CCT3, KIF20B* and *LAMB1*.

## Discussion

The cross-species application of the domestic cat reference presented here takes advantage of the high degree of synteny between Felidae genomes. Previously, cheetah [18] and tiger [20, 22] genomes have shown a high level of conserved synteny and repeat composition when compared with the domestic cat. This similarity is supported by the high alignment quality of all samples included here. Successful alignment of big cat samples and pools resulted in > 99 % coverage of the felCat9 reference assembly at varying sequence depths. Despite differences in phylogenetic distances between Acinonyx and Panthera lineages and domestic cats, the proportion of reads that aligned for each species did not appear to decrease with phylogenetic distance. The high affinity alignment presented here indicates strong genomic conservation within Felidae. This likely reflects the relatively contemporary speciation of modern felids, occurring < 11MYA [46]. Genomic synteny between species provides opportunities to interpret genomic structure and gene function in an evolutionary context. The high degree of synteny observed here is of crucial importance in studying diversity among felid species and mechanisms underlying local adaptation that differ among them [21, 47, 48]. Additionally, the highly conserved synteny of the cat genome with that of humans and other mammalian species has given insight into ancestral genome organisation [49–51], supporting the cat as a valuable biomedical model for heritable human diseases [52–55].

### Estimates of heterozygosity are vulnerable to reference bias

Demographic parameters based on estimates of heterozygosity were inconsistent with previous studies. This may result from small sample sizes, unknown relatedness among samples and the cross-species genome alignment method employed here. While felid genomes may be highly homologous, and previous reports indicate no large-scale chromosomal rearrangements among felids [56], reference sequence bias can substantially impact estimates of heterozygosity and downstream population genomic analyses [57, 58]. Cross-species alignments have been found to bias heterozygosity estimates while correctly measuring population structure [29, 59]. Cheetahs were previously found to have lower rate of heterozygous SNVs when aligned to the draft cheetah assembly (0.0019–0.0021 [18], compared with 0.012 when aligned to the domestic cat reference herein). A similar pattern was also observed for the snow leopard (0.0002 [20] compared with 0.004 herein). In genomic studies, reference-guided variant calling will always be biased toward the properties of the reference genome, rather than those shared across a population. Reference genomes are idiosyncratic, type-specimens and preferential alignment of genomic sequences to the reference alleles, results in underestimating the level of variation in aligned samples from different populations. This problem is demonstrated by the higher SNV frequency in cheetahs (more closely related to the domestic cat), compared with snow leopards and Sumatran tigers. When aligned to its own species’ reference assembly, the cheetah genome displays lower overall SNV frequency and a significantly higher proportion of homozygous stretches than the domestic cat [18]. Any demographic statistics that rely on low frequency variants, may be affected by this bias which likely accounts for high inbreeding coefficients and low heterozygosity observations. Population structure analyses driven by common variants are largely unaffected by reference bias, as MDS showed samples of the same geographic provenance clustered together. Multi-genome alignment techniques that overcome these biases are available in humans, but these resources will likely remain scarce in the context of big cat conservation as they require large sample sets [60–62]. We recommend against using demographic inferences based on cross-species genome alignments to inform conservation management.

### Poolseq is a cost-effective means of SNV discovery

We compared sequencing of individual genomes with sequencing of pooled DNA from multiple (*N* = 4) individuals (poolseq). After quality filtering, pooled samples called more SNV than lower coverage individual WGS (Sumatran tiger and cheetah) and an equivalent number of SNV to high coverage individual WGS (snow leopard). Poolseq can be used to provide a high-quality approach for genotyping the collective genomic profile of a population, comparable to population-level allele frequency estimates of individual WGS [63]. Poolseq is a cost-effective method for assessing population structure and genome-wide patterns of variation [64, 65], but does present statistical challenges in deriving estimates of demographic and other inferences that rely on individual heterozygosity. Estimating allele frequencies from poolseq is vulnerable to experimental noise and bias at a number of protocol stages, from pooling equimolar ratios of DNA [66] through to library construction [67], sequencing and analysis. Poolseq is a less efficient technique for discovering SNVs than individual WGS when coverage is low and sample sizes are small [63]. Previous studies have shown that effects of experimental error are greater when a pool is small (*N* < 10) and sequencing depth is low [68]. Suitably large sample sizes (*N* > 50) are often unfeasible for conservationists working with endangered species, particularly large carnivores that exist in low densities in both the wild and ex situ management settings. To compensate for small pool size (*N* = 4), pools were sequenced at a high coverage, quality-based filtering and MAF-based filtering of SNVs was performed. MAF thresholds for each species group were used to exclude variants present in a heterozygous state in one individual only. This approach can be used to reliably characterise the allele frequency spectrum, however the downside is that attempts to control for high error rates will have excluded low-frequency or rare alleles. As a result of excluding these alleles, we did not include poolseq samples in estimations of demographic parameters. Choosing poolseq under these circumstances is to accept the trade-off of losing information about rare alleles, in favour of a cost-effective estimation of genome-wide allele frequency of a population.

### Genomic signatures into big cat hypercarnivory

Considering our data in the genomic and phenotypic context of the domestic cat has highlighted a suite of defining characteristics of domestication. These characteristics typically include neurological and behavioural changes associated with tameness, a shift toward a polyoestrous reproductive cycle, altered dietary habits and morphological changes [69]. Fixed variants presented here highlight differences in metabolic function, body size and neurological processes in wild and domestic cats. Changes in neural crest-related genes are believed to underlie the evolution of tameness across a range of domesticated species [48, 70–73]. The expression of cell adhesion proteins including cadherins and protocadherins during neural crest cell development is regulated by Wnt signalling [74, 75]. Fixed differences in protocadherin genes is consistent with comparative studies in wildcats (*Felis silvestris*) [48] and domesticated foxes [76]. As hyper-carnivorous ambush predators, cats share physiological traits essential for hunting and endogenous glucose demands [72, 77, 78]. Fixed variants in big cats reveal adaptive physiological functions essential to their evolutionary success as carnivorous species [79, 80]. In cheetahs this included genes involved in spatiotemporal awareness (*HARS1*) [81] and skeletal muscle function (*ACTN3*, *SACS*, *MEGF10, SGCG* and *XIRP1*) [44, 82–84]. Among Sumatran tiger and snow leopards these included unique missense variants in *LCORL*, a gene previously associated with body size in domestic mammals [13, 85, 86]. These results reflect species-specific genetic adaptations associated with hyper-carnivory, highlighting candidate genes underlying species-specific adaptative mechanisms integral to the evolutionary success of big cats. Carnivorous diets are associated with increased metabolism, faster growth rates and higher fecundity [28, 87] and is dependent on an abundance of prey species. Habitat loss and prey depletion threaten the ecological niche occupied by big cats as keystone species [88]. Ongoing genomic studies of these traits can highlight mechanisms by which big cats interact with their ecosystems, complementing ecological studies and serve as an essential component of holistic management plans [89–92].

### Genomic insights into the pathogenesis of diseases affecting big cats

Adding to the ecological complexities of big cat conservation is the impact of infectious and heritable diseases [93, 94]. Captive cheetahs, snow leopards and Sumatran tigers have historically presented a range of infectious and degenerative diseases, while their wild counterparts have remained unaffected [93, 95, 96]. Captive breeding programmes typically operate to maintain within-population genetic diversity, however for many threatened species, population bottlenecks in the wild have resulted in genetically depauperate populations that display impaired fitness and increased susceptibility to infectious diseases [15, 97]. Domestic animals are increasingly used to model complex and simple genetic diseases in humans [52, 54, 98]. The methods employed by these studies, including genome-wide association studies, can be adapted by conservationists studying the genetic basis of heritable diseases. As an example, candidate genes relevant to health and reproductive success studied here can be used to inform studies of the genetic basis of documented conditions of ciliary dysfunction including CVD in Sumatran tigers [95], poor spermatozoan quality in cheetahs [99] and chronic respiratory infections [93, 94], by testing whether any of these variants are associated with clinical findings.

Sumatran tiger cubs in Australian zoos have been reportedly affected by CVD with a heritable component since 1990. Pedigree and segregation analyses suggested an autosomal dominant mode of inheritance with incomplete penetrance of CVD within the Australian population [95]. Stereocilia in the vestibular system play a functional role in spatial navigation and self-motion perception [100] and their dysfunction can cause neurological symptoms, such as those observed in the Sumatran tiger. Genes containing deleterious variants of potential clinical significance identified here included: *SPEF2* previously implicated in primary ciliary dyskinesia [101] and vestibular stereocilia function [102], *HOMER2* a stereociliary scaffolding protein, essential for normal hearing and vestibular function in humans and mice [103, 104] and *PCDH15* implicated in Usher syndrome in humans and balance disorders and deafness in mice [105]. Similar enrichment of ciliary genes was observed in the cheetah with deleterious variants observed in seven genes (*IFT140, CPLANE1, DYNC2H1, CCDC39, CC2DA, RPGRIP1L*) involved in cilium development and structure. Studies in mice and humans have shown defects in ciliary structure and dysmotility are typically present from birth, with affected individuals suffering recurrent respiratory infections and poor fertility [106, 107]. Respiratory infections have long been observed as a significant cause of mortality in captive cheetahs [93, 108]. Data presented here can be used to pinpoint mechanistic bases of ciliary dysfunction in mucociliary clearance of the respiratory tract, paranasal sinuses and middle ear during respiratory infections. Eighteen genes known to cause primary cilia dyskinesia in humans [109] were found to contain missense variants in the cheetah dataset, of these *CCDC39*, *DNAH8 LRRC6* contained deleterious variants. These genes are highly conserved between felids and humans and as such may serve as valuable candidates for understanding the pathogenesis of reproductive, respiratory and vestibular diseases present in big cat populations and aid in improved diagnosis and treatment by veterinarians.

## Conclusions

We have demonstrated the utility of cross-species genome alignments in gaining insights into population structure and functional genomic features in big cat species. The datasets presented here provide a useful resource for future studies into population dynamics, evolutionary history and genetic and disease management of big cats. The high degree of synteny among felid genomes enabled the successful application of the domestic cat reference for visualising population structure, discovering variants associated with adaptive traits, genes under selection and pathogenesis of heritable diseases. Importantly however, this method is limited in its capacity to adequately quantify heterozygosity and low-frequency variants. Poolseq proved a low-cost method for genotypic profiling of each species. This cross-species method of variant discovery provides genomic context for identifying annotated gene regions essential to understanding the genomic landscape underpinning traits and diseases that can be used to improve conservation outcomes.

## Methods

### Animals and DNA sequencing

Whole genome sequences for seven Sumatran tigers, six cheetahs and one snow leopard were downloaded from Sequence Read Archive (SRA) in fastq format (Table S9). Sumatran tiger samples originated from American and Indonesian facilities, the snow leopard individual originated from Korea and Cheetah samples originated from free-ranging individuals in Nambia and Tanzania. Whole blood samples from four snow leopards, four cheetahs and four Sumatran tigers currently housed in Australian zoos were collected as a part of routine health examinations by registered veterinarians employed by each zoo and submitted to the University for infectious disease screening. Individuals comprising each species pool were chosen from a broader cohort based on pairwise relatedness (siblings and parent-offspring pairs were excluded) and individual inbreeding coefficients (F_IS_) calculated using International Studbook data. Genomic DNA was isolated from whole blood by phenol-chloroform extraction and pooled by species in equimolar ratios (4 individuals/species pool). Library preparation and whole genome sequencing of species pools was performed by Ramaciotti Centre for Genomics, University of New South Wales (Kensington, Australia). Illumina paired-end libraries were prepared and sequenced on the Illumina HiSeq 2000 platform with 150 bp paired-end reads.

### Reference genome alignment, variant calling and filtering

All samples were aligned to the felCat9 reference assembly using Burrows Wheeler Aligner algorithm, BWA-mem [110]. Aligned reads were sorted using Samtools 1.9 [111]. Base quality score recalibration was performed using Genome Analysis ToolKit (GATK) [112]. Summary statistics of alignments were collected using Samtools stats function. GATK best practices [113] were used for SNV and short indel calling. For each sample, GATK’s HaplotypeCaller tool was used to call variants from the recalibrated bam file. GVCF files containing unfiltered SNVs and short indel calls for all sites were then submitted for joint genotyping with GATK’s GenotypeGVCF tool. A VCF was generated for each species cohort and then passed to VariantFiltration tool for hard filtering according to GATK recommendations: QUAL > 40.0, QD > 2.0, MQ > 50.0, FS < 50 and then to SelectVariants tool to remove indel calls. Summary statistics of quality filtered VCF datasets and diversity estimates for individuals and species cohorts including: nucleotide diversity (π), TsTv ratio, and individual heterozygosity (H_s_) were performed using VCFTools –site-pi, --tstv and –het flags [114]. Species-specific SNVs (fixed) were collected from hard-filtered species cohort VCFs using minor allele frequency (MAF) thresholds (Table 2) with BCFtools view function [115]. Population structure within species was detected by MDS using PLINK [116] with MAF-filtered SNV datasets. To examine whether any individuals were closely related, relatedness among individuals in each species cohort were calculated using VCFTools –relatedness2 function.

### Variant annotation and gene enrichment analysis

Variant datasets were annotated using Ensembl Variant Effect Predictor (VEP) tool [117] and the NCBI annotation release 104 of the felCat9 genome build. Gene annotation and enrichment analyses were performed for fixed and MAF-filtered datasets on protein change variants annotated by VEP with the following impact terms: missense_variant, start_lost, stop_gained, stop_lost, stop_retained_variant, splice_acceptor_variant, splice_donor_variant. Genes with an accelerated rate of non-synonymous to synonymous substitutions (d_N_/d_S_) and nucleotide diversity of non-synonymous to synonymous coding sites (π_N_/π_S_) within each species were calculated using SNPGenie v1.2 [118]. Enrichment analysis of GO terms and pathways was performed using gPofiler [119]. The gProfiler gOSt function gathers functional annotation terms from various annotation sources including gene ontology terms, biological pathways, protein databases and human phenotype ontology. Enrichment terms were considered significant if they passed a significance threshold of *P* < 0.001, corrected for multiple testing. This was done for fixed variant sets for each species to highlight genes of potential significance in felid evolution. It was also performed for MAF-filtered SNV datasets to highlight genes of potential significance within each species. GO enrichment output of fixed and MAF-filtered datasets from gProfiler were reduced to their most specific GOterms using REVIGO [120]. Network pathway enrichment analysis was performed using WebGestalt [121] overrepresentation analysis and EnrichNet [122] to identify network interconnectivity score (XD score) and overlap-based enrichment score (Fisher’s exact test) of genes containing fixed variants and known phenotypic annotations. Phenotypic annotation of genes containing fixed SNVs for each species to the OMIM [123] database was performed using Ensembl’s BioMart [124]. A list of genes previously associated with body size in domestic species was collected from the literature (Table S10) and cross-searched using fixed variant datasets in all big cat species. MEGA-X software [125] was used to perform multiple sequence alignment of *LCORL* protein, previously implicated in body size variation among mammals. *LCORL* protein sequences for domestic dog (*Canis lupus familiaris*), domestic cat, horse (*Equus caballus*), cow (*Bos taurus*) and lion were downloaded from Ensembl and aligned to consensus FASTA sequences of cheetah, Sumatran tiger and snow leopard. Multiple sequence alignment was created using the Muscle algorithm.

To identify genes potentially implicated in the reproductive success and overall health of captive-bred big cats, a list of clinically relevant genes was curated using GO annotation terms with AmiGO2 [126] in humans, dogs, pigs, cats, rats and mice (Table S11). This list comprised genes implicated in a list of heritable conditions affecting captive-bred cheetahs, snow leopard and Sumatran tigers, compiled from literature (Table S12) and genes implicated in reproduction, immunity and embryonic development. Variants in these genes were collected from each species cohort MAF-filtered VCF file and submitted for annotation analysis using GOnet [127] and Web-Gestalt using over-representation analysis and disease OMIM and GLAD4U [128] functional databases. High impact variants in these genes were included if they had been annotated as ‘deleterious’ by VEP.

## Supplementary Information

**Additional file 1: Table S1.** Sequencing and alignment performance for all individual samples and species pools. Cheetah_pool, tiger_pool and snowleopard_pool refer to species pools consisting of 4 multiplexed individuals each. **Figure S1.** Mapped and total reads across WGS samples and pools. Total number of mapped reads (black) includes singletons and pairs. Number of paired reads mapped indicated by the grey line. Tiger_zoo, snowleopard_zoo and cheetah_zoo refer to multiplexed pool samples. **Figure S2.** Plots of genome coverage for each sample .bam file aligned to the felCat9 reference assembly for a. cheetah, b. Sumatran tiger and c. snow leopard cohorts. On each panel, the key indicates coloured line of each sample and their sequencing depth in brackets. **Table S2.** Functional annotation of all fixed and within-species SNPs for each species. **Table S3a.** Top 20 gene ontology terms (GOterms) enriched for species-specific SNV within cheetahs. *P*-values were adjusted for multiple testing using Benjamini-Hochberg false discovery rate. **Table S3b.** Top 20 gene ontology terms (GOterms) enriched for species specific SNVs within Sumatran tigers. *P*-values were adjusted for multiple testing using Benjamini-Hochberg false discovery rate. **Table S4. **Genes under positive selection identified as those displaying elevated π_N_π_S_ratios across all three species. **Table S5a.** Top 20 gene ontology terms (GOterms) enriched across fixed SNVs in snow leopards. *P*-values were adjusted for multiple testing using Benjamini-Hochberg FDR (false discovery rate). **Table S5b.** Top 20 gene ontology terms (GOterms) enriched across fixed SNVs in Sumatran tigers. *P*-values were adjusted for multiple testing using Benjamini-Hochberg FDR (false discovery rate). **Table S6.** Multi-species alignment of *LCORL* (ENSFCAG00000029474) revealed Panthera-specific conservation of six missense variants. Cheetah, snow leopard and Sumatran tiger refer to samples aligned to the domestic cat (felCat9) reference assembly. Protein positions are reported relative to the Ensembl transcript ENSFCAT00000081895.1. **Table S7. **Protocadherin genes containing fixed non-synonymous SNVs common to all big cat species relative to the domestic cat (felCat9) reference assembly. **Table S8.** Genes containing deleterious SNVs implicated in heritable conditions affecting big cats grouped by species. **Figure S3a.** Gene ontology annotation of clinically significant GOterms in cheetahs. Deleterious variants were observed in 201 genes included in custom list of GO terms relevant to known heritable conditions, immune and reproductive function. These genes were annotated for terms relevant to reproductive and immune function. Network interaction graph produced by GOnet (https://tools.dice-database.org/GOnet/). **Figure S3b.** Gene ontology annotation of clinically significant GOterms in Sumatran tigers. Deleterious variants were observed in 44 genes included in custom list of GO terms relevant to known heritable conditions, immune and reproductive function. These genes were annotated for terms relevant to cilium structure. Network interaction graph produced by GOnet (https://tools.dice-database.org/GOnet/). **Table S9.** Samples downloaded from Sequence Read Archive (SRA) comprised six cheetahs, one snow leopard and seven Sumatran tigers. **Table S10.**Genes associated with size in domestic species identified from a literature search. **Table S11.** Selected GO enrichment terms used to classify deleterious variants potentially implicated in reproductive success and overall health of captive bred big cats. **Table S12.** Known heritable conditions affecting captive bred big cat species and relevant GO terms.

## Data Availability

Sequence data for cheetah, Sumatran tiger and snow leopard individual samples is available on NCBI Sequence Read Archive. Cheetah samples: SRR2737540, SRR2737541, SRR2737542, SRR2737543, SRR2737544, SRR2737545 were deposited under BioProject no. PRJNA297824. Sumatran tiger samples: SRR7152379, SRR7152382, SRR7152383, SRR7152384, SRR7152385, SRR7152386, SRR7152388 were deposited under BioProject no. PRJNA437782. Snow leopard sample SRR836372 was deposited under BioProject no. PRJNA182708. Final VCF files for cheetahs is available at 10.6084/m9.figshare.12996920, for Sumatran tigers is available at 10.6084/m9.figshare.12996947 and for snow leopards is available at 10.6084/m9.figshare.12996977.
