# Supplementary material for: Exploiting genomic synteny in Felidae: cross-species genome alignments and SNV discovery can aid conservation management
Source: BMC Genomics. 2021 Aug 6;22:601. doi: 10.1186/s12864-021-07899-2 (PMC8348863; doi:10.1186/s12864-021-07899-2)
Supplement: Supplementary file 1 — Additional file 1: Table S1. Sequencing and alignment performance for all individual samples and species pools. Cheetah_pool, tiger_pool and snowleopard_pool refer to species pools consisting of 4 multiplexed individuals each. Figure S1. Mapped and total reads across WGS samples and pools. Total number of mapped reads (black) includes singletons and pairs. Number of paired reads mapped indicated by the grey line. Tiger_zoo, snowleopard_zoo and cheetah_zoo refer to multiplexed pool samples. Figure S2. Plots of genome coverage for each sample .bam file aligned to the felCat9 reference assembly for a. cheetah, b. Sumatran tiger and c. snow leopard cohorts. On each panel, the key indicates coloured line of each sample and their sequencing depth in brackets. Table S2. Functional annotation of all fixed and within-species SNPs for each species. Table S3a. Top 20 gene ontology terms (GOterms) enriched for species-specific SNV within cheetahs. P-values were adjusted for multiple testing using Benjamini-Hochberg false discovery rate. Table S3b. Top 20 gene ontology terms (GOterms) enriched for species specific SNVs within Sumatran tigers. P-values were adjusted for multiple testing using Benjamini-Hochberg false discovery rate. Table S4. Genes under positive selection identified as those displaying elevated πNπSratios across all three species. Table S5a. Top 20 gene ontology terms (GOterms) enriched across fixed SNVs in snow leopards. P-values were adjusted for multiple testing using Benjamini-Hochberg FDR (false discovery rate). Table S5b. Top 20 gene ontology terms (GOterms) enriched across fixed SNVs in Sumatran tigers. P-values were adjusted for multiple testing using Benjamini-Hochberg FDR (false discovery rate). Table S6. Multi-species alignment of LCORL (ENSFCAG00000029474) revealed Panthera-specific conservation of six missense variants. Cheetah, snow leopard and Sumatran tiger refer to samples aligned to the domestic cat (felCat9) reference assembly. Protein positio [file 12864_2021_7899_MOESM1_ESM.docx]

**Table S1: Sequencing and alignment performance for all individual samples and species pools. Cheetah_pool, tiger_pool and snowleopard_pool refer to species pools consisting of 4 multiplexed individuals each.**


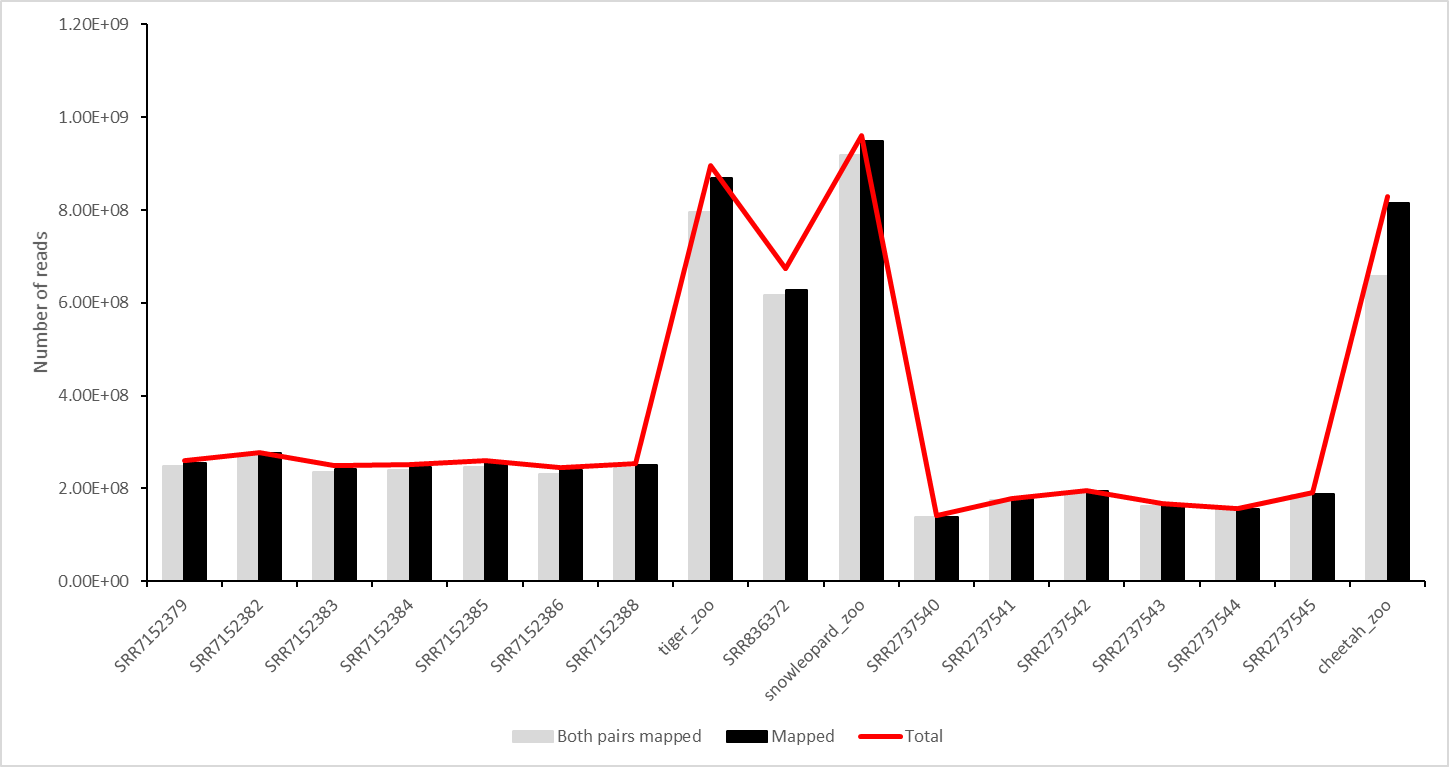


**Figure S1: mapped and total reads across WGS samples and pools.** Total number of mapped reads (black) includes singletons and pairs. Number of paired reads mapped indicated by the grey line. Tiger_zoo, snowleopard_zoo and cheetah_zoo refer to multiplexed pool samples.


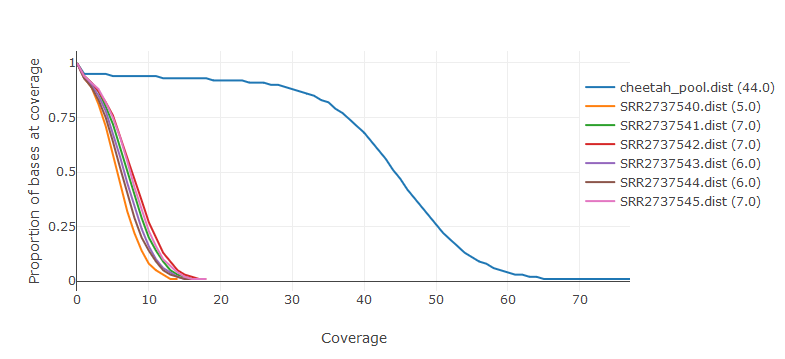

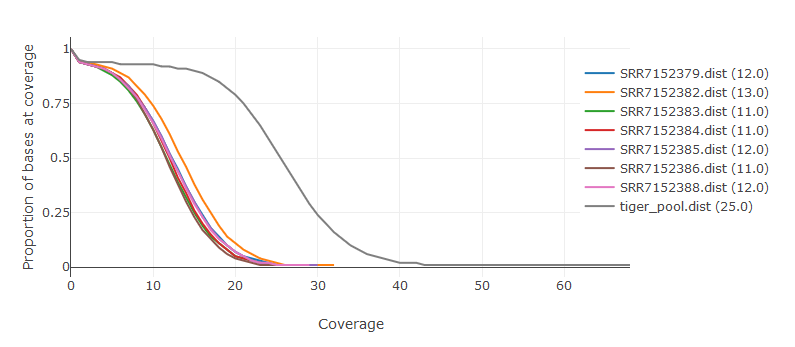

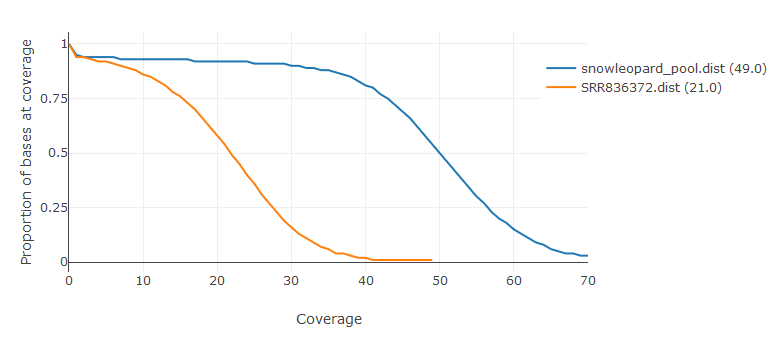


**Figure S2: Plots of genome coverage for each sample bam file aligned to the felCat9 reference assembly for a.** cheetah, **b.** Sumatran tiger and **c.** snow leopard cohorts. On each panel, the key indicates coloured line of each sample and their sequencing depth in brackets.

**a**

**b**

**c**

**Table S2: Functional annotation of all fixed and within-species SNPs for each species**

**Table S3a: Top 20 gene ontology terms (GOterms) enriched for species-specific SNV within cheetahs. P-values were adjusted for multiple testing using Benjamini-Hochberg false discovery rate.**

| **GOtermID** | **GOterm name** | **Padj** |
| --- | --- | --- |
| GO:0005515 | protein binding | 3.23E-23 |
| GO:0051179 | localization | 6.31E-22 |
| GO:0032502 | developmental process | 7.91E-17 |
| GO:0048856 | anatomical structure development | 1.45E-16 |
| GO:0007275 | multicellular organism development | 2.97E-15 |
| GO:0005488 | binding | 5.64E-15 |
| GO:0120025 | plasma membrane bounded cell projection | 3.26E-14 |
| GO:0042995 | cell projection | 3.77E-14 |
| GO:0048731 | system development | 2.71E-13 |
| GO:0043167 | ion binding | 8.85E-12 |
| GO:0005856 | cytoskeleton | 9.20E-12 |
| GO:0006928 | movement of cell or subcellular component | 3.60E-11 |
| GO:0051234 | establishment of localization | 5.54E-11 |
| GO:0097367 | carbohydrate derivative binding | 7.83E-11 |
| GO:0030554 | adenyl nucleotide binding | 1.25E-10 |
| GO:0032559 | adenyl ribonucleotide binding | 1.65E-10 |
| GO:0048869 | cellular developmental process | 2.48E-10 |
| GO:0043168 | anion binding | 6.42E-10 |
| GO:0036094 | small molecule binding | 1.27E-09 |
| GO:0005524 | ATP binding | 1.56E-09 |

**Table S3b: Top 20 gene ontology terms (GOterms) enriched for species specific SNVs within Sumatran tigers. P-values were adjusted for multiple testing using Benjamini-Hochberg false discovery rate.**

| **GOtermID** | **GOterm name** | **Padj** |
| --- | --- | --- |
| GO:0005515 | protein binding | 6.01E-95 |
| GO:0003824 | catalytic activity | 9.19E-56 |
| GO:0043168 | anion binding | 2.36E-52 |
| GO:0036094 | small molecule binding | 4.08E-40 |
| GO:1901265 | nucleoside phosphate binding | 1.18E-39 |
| GO:0000166 | nucleotide binding | 1.47E-39 |
| GO:0097367 | carbohydrate derivative binding | 2.59E-38 |
| GO:0030554 | adenyl nucleotide binding | 2.74E-37 |
| GO:0032559 | adenyl ribonucleotide binding | 7.81E-37 |
| GO:0017076 | purine nucleotide binding | 1.74E-34 |
| GO:0032553 | ribonucleotide binding | 1.81E-34 |
| GO:0005524 | ATP binding | 2.87E-34 |
| GO:0032555 | purine ribonucleotide binding | 7.19E-34 |
| GO:0035639 | purine ribonucleoside triphosphate binding | 1.09E-31 |
| GO:0019899 | enzyme binding | 5.86E-29 |
| GO:0016787 | hydrolase activity | 7.10E-28 |
| GO:0016772 | transferase activity, transferring phosphorus-containing groups | 9.10E-28 |
| GO:0016301 | kinase activity | 1.84E-27 |
| GO:0016773 | phosphotransferase activity, alcohol group as acceptor | 2.25E-27 |
| GO:0008092 | cytoskeletal protein binding | 5.09E-26 |

**Table S4: Genes under positive selection identified as those displaying elevated π_N_π_S_ ratios across all three species.**

| **Species** | **Gene name** | **Gene symbol** | **Gene ID** | **π_N_π_S_** |
| --- | --- | --- | --- | --- |
| Sumatran tiger | Alpha 1-3-galactosyltransferase | LOC101087791 | ENSFCAG00000025873 | 3.04 |
|  | Vomeronasal type-1 receptor | LOC101091239 | ENSFCAG00000025070 | 1.45 |
|  | none | none | ENSFCAG00000053257 | 1.22 |
|  | none | none | ENSFCAG00000051471 | 1.16 |
|  | Olfactory receptor 5H2 | LOC101089384 | ENSFCAG00000049952 | 1.16 |
|  | none | none | ENSFCAG00000045227 | 1.13 |
| Snow leopard | none | LOC101089645 | ENSFCAG00000040741 | 1.47 |
| Cheetah | sidekick cell adhesion molecule 1 | SDK1 | ENSFCAG00000012315 | 77.66 |
|  | N-terminal EF-hand calcium binding protein 2 | NECAB2 | ENSFCAG00000026632 | 18.62 |
|  | membrane spanning 4-domains A7 | MS4A7 | ENSFCAG00000007252 | 15.65 |
|  | epoxide hydrolase 1 | EPHX1 | ENSFCAG00000006965 | 5.62 |
|  | olfactory receptor 2A1/2A42-like | LOC101085032 | ENSFCAG00000043770 | 2.87 |
|  | RAB44 | RAB44 | ENSFCAG00000023379 | 2.79 |
|  | none | none | ENSFCAG00000029091 | 2.27 |
|  | G2 and S-phase expressed 1 | GTSE1 | ENSFCAG00000006532 | 2.26 |
|  | dynein axonemal heavy chain 2 | DNAH2 | ENSFCAG00000009626 | 2.12 |
|  | synaptotagmin like 2 | SYTL2 | ENSFCAG00000029903 | 2.06 |
|  | toll like receptor 3 | TLR3 | ENSFCAG00000031090 | 1.91 |
|  | 2'-5'-oligoadenylate synthetase 3 | OAS3 | ENSFCAG00000000320 | 1.87 |
|  | Fc receptor like 4 | FCRL4 | ENSFCAG00000038609 | 1.86 |
|  | adaptor related protein complex 3 subunit sigma 1 | AP3S1 | ENSFCAG00000029920 | 1.82 |
|  | none | none | ENSFCAG00000051726 | 1.78 |
|  | ribosome biogenesis protein NSA2 homolog | LOC101094823 | ENSFCAG00000025379 | 1.73 |
|  | BTB domain containing 16 | BTBD16 | ENSFCAG00000003652 | 1.72 |
|  | olfactory receptor 151-like | LOC101084218 | ENSFCAG00000033436 | 1.71 |
|  | dynein axonemal heavy chain 6 | DNAH6 | ENSFCAG00000000768 | 1.68 |
|  | none | none | ENSFCAG00000052912 | 1.66 |
|  | none | none | ENSFCAG00000049647 | 1.63 |
|  | migration and invasion inhibitory protein | MIIP | ENSFCAG00000000077 | 1.59 |
|  | microtubule associated serine/threonine kinase family member 4 | MAST4 | ENSFCAG00000025450 | 1.46 |
|  | none | none | ENSFCAG00000048579 | 1.43 |
|  | sortilin related VPS10 domain containing receptor 2 | SORCS2 | ENSFCAG00000015288 | 1.36 |
|  | maestro heat like repeat family member 8 | MROH8 | ENSFCAG00000011925 | 1.34 |
|  | olfactory receptor-like protein OLF3 | LOC101101377 | ENSFCAG00000040453 | 1.27 |
|  | coiled-coil domain containing 136 | CCDC136 | ENSFCAG00000043434 | 1.25 |
|  | adhesion G protein-coupled receptor G6 | ADGRG6 | ENSFCAG00000024629 | 1.24 |
|  | olfactory receptor 5V1-like | LOC101095034 | ENSFCAG00000027476 | 1.23 |
|  | cortactin | CTTN | ENSFCAG00000004767 | 1.22 |
|  | sarcoglycan gamma | SGCG | ENSFCAG00000033144 | 1.2 |
|  | cytoskeleton associated protein 2 | CKAP2 | ENSFCAG00000010021 | 1.19 |
|  | polo like kinase 5 | PLK5 | ENSFCAG00000001784 | 1.18 |
|  | putative olfactory receptor 5AK3 | LOC101086178 | ENSFCAG00000027084 | 1.16 |
|  | C2 domain containing 3 centriole elongation regulator | C2CD3 | ENSFCAG00000028884 | 1.14 |
|  | none | none | ENSFCAG00000024754 | 1.1 |
|  | xin actin binding repeat containing 1 | XIRP1 | ENSFCAG00000013533 | 1.1 |
|  | leucine rich repeat containing 66 | LRRC66 | ENSFCAG00000042278 | 1.09 |
|  | none | none | ENSFCAG00000035027 | 1.09 |
|  | none | none | ENSFCAG00000053257 | 1.08 |
|  | ribokinase | RBKS | ENSFCAG00000003303 | 1.08 |
|  | hemicentin 2 | HMCN2 | ENSFCAG00000030267 | 1.08 |
|  | fms related tyrosine kinase 4 | FLT4 | ENSFCAG00000001882 | 1.07 |
|  | none | none | ENSFCAG00000040052 | 1.05 |
|  | proline rich 14 like | PRR14L | ENSFCAG00000029759 | 1.04 |
|  | olfactory receptor 51G2-like | LOC101092885 | ENSFCAG00000025216 | 1.04 |
|  | RAB11 family interacting protein 1 | RAB11FIP1 | ENSFCAG00000028568 | 1.03 |
|  | anoctamin 7 | ANO7 | ENSFCAG00000010453 | 1.03 |
|  | none | none | ENSFCAG00000003805 | 1.03 |
|  | PBX homeobox interacting protein 1 | PBXIP1 | ENSFCAG00000001344 | 1.03 |
|  | EF-hand calcium-binding domain-containing protein 3 | LOC102899173 | ENSFCAG00000003882 | 1.02 |
|  | ATR serine/threonine kinase | ATR | ENSFCAG00000018078 | 1 |

**Table S5a: Top 20 gene ontology terms (GOterms) enriched across fixed SNVs in snow leopards. P-values were adjusted for multiple testing using Benjamini-Hochberg FDR (false discovery rate).**

| **GOtermID** | **GOterm name** | **Padj** |
| --- | --- | --- |
| GO:0005515 | protein binding | 4.33E-72 |
| GO:0032502 | developmental process | 1.05E-58 |
| GO:0048856 | anatomical structure development | 5.03E-58 |
| GO:0005488 | binding | 2.69E-52 |
| GO:0005737 | cytoplasm | 1.15E-50 |
| GO:0007275 | multicellular organism development | 6.02E-50 |
| GO:0048731 | system development | 1.39E-48 |
| GO:0043227 | membrane-bounded organelle | 2.47E-45 |
| GO:0005622 | intracellular | 3.96E-43 |
| GO:0048518 | positive regulation of biological process | 2.30E-39 |
| GO:0043231 | intracellular membrane-bounded organelle | 2.54E-37 |
| GO:0048522 | positive regulation of cellular process | 1.82E-35 |
| GO:0048513 | animal organ development | 3.84E-35 |
| GO:0048519 | negative regulation of biological process | 7.14E-35 |
| GO:0048869 | cellular developmental process | 8.16E-35 |
| GO:0030154 | cell differentiation | 2.10E-34 |
| GO:0043226 | organelle | 9.86E-34 |
| GO:0051179 | localization | 2.55E-33 |
| GO:0048523 | negative regulation of cellular process | 2.17E-31 |
| GO:0051239 | regulation of multicellular organismal process | 1.21E-30 |

**Table S5b: Top 20 gene ontology terms (GOterms) enriched across fixed SNVs in Sumatran tigers. P-values were adjusted for multiple testing using Benjamini-Hochberg FDR (false discovery rate).**

| **GOtermID** | **GOterm name** | **Padj** |
| --- | --- | --- |
| GO:0005515 | protein binding | 6.01E-95 |
| GO:0003824 | catalytic activity | 9.19E-56 |
| GO:0043168 | anion binding | 2.36E-52 |
| GO:0036094 | small molecule binding | 4.08E-40 |
| GO:1901265 | nucleoside phosphate binding | 1.18E-39 |
| GO:0000166 | nucleotide binding | 1.47E-39 |
| GO:0097367 | carbohydrate derivative binding | 2.59E-38 |
| GO:0030554 | adenyl nucleotide binding | 2.74E-37 |
| GO:0032559 | adenyl ribonucleotide binding | 7.81E-37 |
| GO:0017076 | purine nucleotide binding | 1.74E-34 |
| GO:0032553 | ribonucleotide binding | 1.81E-34 |
| GO:0005524 | ATP binding | 2.87E-34 |
| GO:0032555 | purine ribonucleotide binding | 7.19E-34 |
| GO:0035639 | purine ribonucleoside triphosphate binding | 1.09E-31 |
| GO:0019899 | enzyme binding | 5.86E-29 |
| GO:0016787 | hydrolase activity | 7.10E-28 |
| GO:0016772 | transferase activity, transferring phosphorus-containing groups | 9.10E-28 |
| GO:0016301 | kinase activity | 1.84E-27 |
| GO:0016773 | phosphotransferase activity, alcohol group as acceptor | 2.25E-27 |
| GO:0008092 | cytoskeletal protein binding | 5.09E-26 |

# Table S6: Multi-species alignment of *LCORL* (ENSFCAG00000029474) revealed *Panthera*-specific conservation of six missense variants. Cheetah, snow leopard and Sumatran tiger refer to samples aligned to the domestic cat (felCat9) reference assembly. Protein positions are reported relative to the Ensembl transcript ENSFCAT00000081895.1

| **Genomic position** | **B1:194193762** | **B1:194194515** | **B1:194194793** | **B1:194194989** | **B1:194195030** | **B1:194196732** | **B1:194196965** | **B1:194197053** | **B1:194197775** | **B1:194197785** | **B1:194197940** |
| --- | --- | --- | --- | --- | --- | --- | --- | --- | --- | --- | --- |
| **Nucleotide variant** | A/G | G/A | G/C | C/T | T/C | T/C | C/T | G/A | T/C | G/A | A/T |
| **protein position** | 393 | 644 | 737 | 802 | 816 | 1383 | 1461 | 1490 | 1731 | 1734 | 1786 |
| **Cat amino acid variant** | N/S | R/K | A/P | A/V | S/P | L/P | R/C | R/Q | F/L | R/K | N/Y |
| *Cheetah* | **S** | **K** | **A** | **A** | **S** | **L** | **R** | **R** | **F** | **R** | **N** |
| *Snow leopard* | **S** | **K** | **P** | **V** | **P** | **P** | **C** | **Q** | **L** | **K** | **Y** |
| *Sumatran tiger* | **S** | **K** | **P** | **V** | **P** | **P** | **C** | **Q** | **L** | **K** | **Y** |
| *Lion* | **S** | **K** | **P** | **V** | **P** | **P** | **C** | **R** | **L** | **K** | **Y** |
| *Domestic cat* | **N** | **R** | **A** | **A** | **S** | **L** | **R** | **R** | **F** | **R** | **N** |
| *Domestic dog* | **S** | **K** | **A** | **T** | **S** | **T** | **H** | **R** | **L** | **R** | **N** |
| *Cow* | **N** | **K** | **A** | **T** | **S** | **A** | **H** | **Q** | **L** | **R** | **N** |
| *Horse* | **H** | **K** | **A** | **T** | **P** | **A** | **H** | **R** | **L** | **R** | **N** |

**Table S7: Protocadherin genes containing fixed non-synonymous SNVs common to all big cat species relative to the domestic cat (felCat9) reference assembly.**

| **Gene** | **Transcript ID** | **gDNA** | **Reference allele (felCat9)** | **Big cat allele** | **Amino acid** | **Consequence** |
| --- | --- | --- | --- | --- | --- | --- |
| *PCDHAC2* | ENSFCAT00000078597.1 | A1:119042691 | C | A | Q/K | Missense |
| *PCDHB1* | ENSFCAT00000003688.4 | A1:119266039 | G | A | A/T | Missense |
| *PCDHB4* | ENSFCAT00000001366.6 | A1:119316197 | A | G | S/N | Missense |
|  |  | A1:119316209 | T | G | W/L | Missense |
| *PCDHB5* | ENSFCAT00000065939.1 | A1:119316539 | C | T | S/P | Missense |
|  |  | A1:119317400 | A | G | V/I | Missense |
| *PCDHB13* | ENSFCAT00000045876.3 | A1:119350686 | A | G | R/Q | Missense |
| *PCDHGB1* | ENSFCAT00000049228.3 | A1:119479145 | T | G | V/L | Missense |
|  |  | A1:119480423 | A | C | P/T | Missense |
| *PCDHGA6* | ENSFCAT00000028046.4 | A1:119504728 | C | T | - | Splice donor |

**Table S8: Genes containing deleterious SNVs implicated in heritable conditions affecting big cats grouped by species.**

| **Sumatran tiger** |  |  |  |  |
| --- | --- | --- | --- | --- |
| *APOB* | *CEP350* | *GALNTL5* | *MAST2* | *PHACTR4* |
| *ATAD5* | *CEP72* | *GAPDH* | *MGAT4D* | *SPACA1* |
| *ATR* | *DDIAS* | *GCNT3* | *MROH2B* | *SPAG6* |
| *BTBD18* | *DNAH7* | *HAUS3* | *MSH6* | *SPATA7* |
| *CALR3* | *DRD5* | *HAUS6* | *MT-ND4* | *SPEF2* |
| *CCP110* | *DUSP13* | *HOMER2* | *NSRP1* | *TMF1* |
| *CCT3* | *DYNC2H1* | *IL2RA* | *PAQR5* | *ZFX* |
| *CENPJ* | *ENO1* | *LRGUK* | *PCDH15* | *ZP2* |
| *CEP19* | *FHDC1* | *LRRK2* | *PCM1* |  |
| **Snow leopard** |  |  |  |  |
| *ADAM29* |  |  |  |  |
| *ADCY10* |  |  |  |  |
| *CCNYL1* |  |  |  |  |
| *CCT3* |  |  |  |  |
| *KIF20B* |  |  |  |  |
| *LAMB1* |  |  |  |  |
| **Cheetah** |  |  |  |  |
| *ABL1* | *CABYR* | *CPLANE1* | *FETUB* | *KLRK1* |
| *ACAN* | *CACNA1S* | *CR2* | *FGF18* | *KNL1* |
| *ADAM20* | *CAPN2* | *CRTAP* | *FLT1* | *KRT9* |
| *ADAM29* | *CAST* | *CST11* | *FRAS1* | *LAMA3* |
| *ADAR* | *CC2D2A* | *CTSH* | *FREM1* | *LRGUK* |
| *ADGRF4* | *CCDC33* | *CUL3* | *FREM2* | *LRIG3* |
| *ADGRF5* | *CCDC39* | *DDO* | *GALNTL5* | *LRP1B* |
| *AHI1* | *CCDC42* | *DLC1* | *GGN* | *LRP2* |
| *ALMS1* | *CCDC62* | *DNAAF1* | *GINS4* | *LRRK2* |
| *AMER1* | *CCN1* | *DNAH1* | *GPR31* | *LUM* |
| *AMH* | *CD19* | *DNAH2* | *H1-6* | *MAP7* |
| *APOB* | *CD1B* | *DNAH8* | *HERPUD2* | *MASTL* |
| *AR* | *CDH23* | *DNAJA1* | *HMX2* | *MATN3* |
| *ARID4B* | *CDK16* | *DNALI1* | *HSP90AA1* | *MBTPS2* |
| *ASXL2* | *CENPU* | *DRC7* | *HSPA8* | *MEIKIN* |
| *ATP2B4* | *CEP290* | *DUOX2* | *HSPE1-RS1* | *MMP13* |
| *ATP8B3* | *CFAP44* | *DUSP13* | *HSPG2* | *MORC1* |
| *ATR* | *CFAP69* | *DYNC2H1* | *HTR2B* | *MROH2B* |
| *AURKA* | *CLEC4G* | *EFCAB9* | *IFT140* | *MVK* |
| *BARD1* | *CLEC7A* | *EHMT2* | *IL4R* | *NCAPG2* |
| *BRCA1* | *CLOCK* | *EPO* | *INVS* | *NES* |
| *C2CD3* | *CLUAP1* | *ERBIN* | *IQCG* | *MYCBPAP* |
| *C5* | *CNTRL* | *FABP9* | *KDM6A* | *MYO3B* |
| *C6* | *COMT* | *FCRL6* | *KIAA1217* | *NAGLU* |
| *C8A* | *CP* | *FDPS* | *KLHL12* | *NBN* |
| *NODAL* | *PRSS55* | *SPATA5* | *TPPP2* | *TEX14* |
| *NUP210L* | *RAD21L1* | *SPATA6* | *TRAF3IP1* | *TEX15* |
| *OFD1* | *RBBP6* | *SPEF2* | *TRPC6* | *TLR8* |
| *OR7C1* | *RECK* | *SPTBN4* | *TTC21A* | *TP53BP1* |
| *OVOL1* | *RIPK3* | *SRD5A2* | *TTK* | *SLC38A10* |
| *PALLD* | *ROPN1L* | *STPG4* | *TUT7* | *SLC9B2* |
| *PANX1* | *RPGRIP1L* | *SUSD4* | *USP9X* | *SOS1* |
| *PAQR7* | *RTN4* | *SYNE1* | *USPL1* | *SPACA1* |
| *PAX8* | *SAXO1* | *TACR2* | *UTP25* | *PLPP4* |
| *PCDH15* | *SCUBE2* | *TBC1D21* | *WDR38* | *POU5F1* |
| *PCDH8* | *SERPINA5* | *TCOF1* | *WDR66* | *PRDX3* |
| *PCDHGA4* | *SERPINH1* | *TDRD1* | *WNK1* | *PRKDC* |
| *PHGDH* | *SHBG* | *TEKT4* | *ZAN* | *ZFX* |
| *PHLDB1* | *SIK3* | *TEPP* | *ZEB2* | *TET1* |
| *PLEKHA1* | *SIRT2* | *TESMIN* | *ZFPM1* | *SLC26A3* |
| *PLK4* |  |  |  |  |


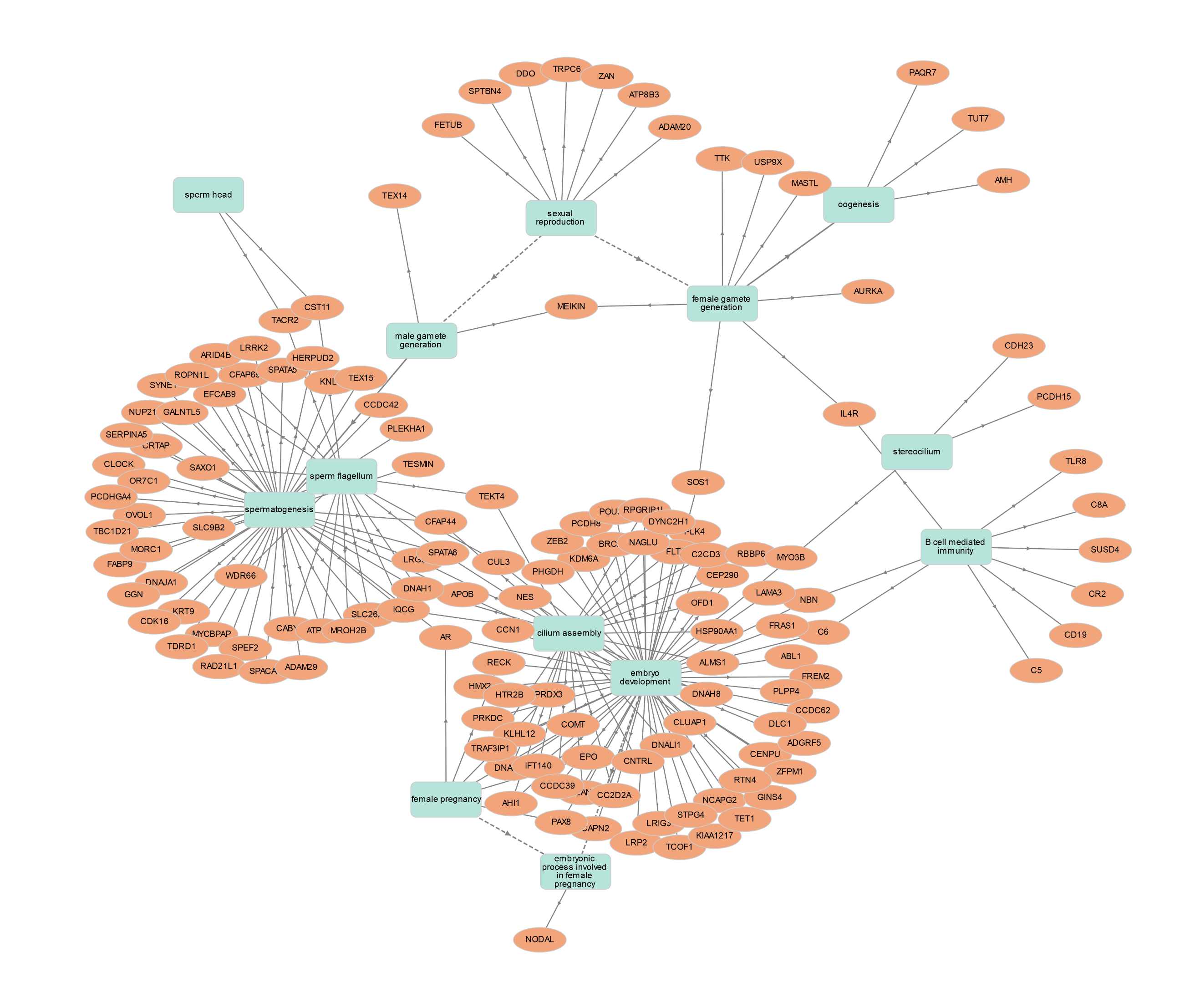


**Figure S3a: Gene ontology annotation of clinically significant GOterms in cheetahs. Deleterious variants were observed in 201 genes included in custom list of GO terms relevant to known heritable conditions, immune and reproductive function. These genes were annotated for terms relevant to reproductive and immune function. Network interaction graph produced by GOnet (**[**https://tools.dice-database.org/GOnet/**](https://tools.dice-database.org/GOnet/)**)**

**
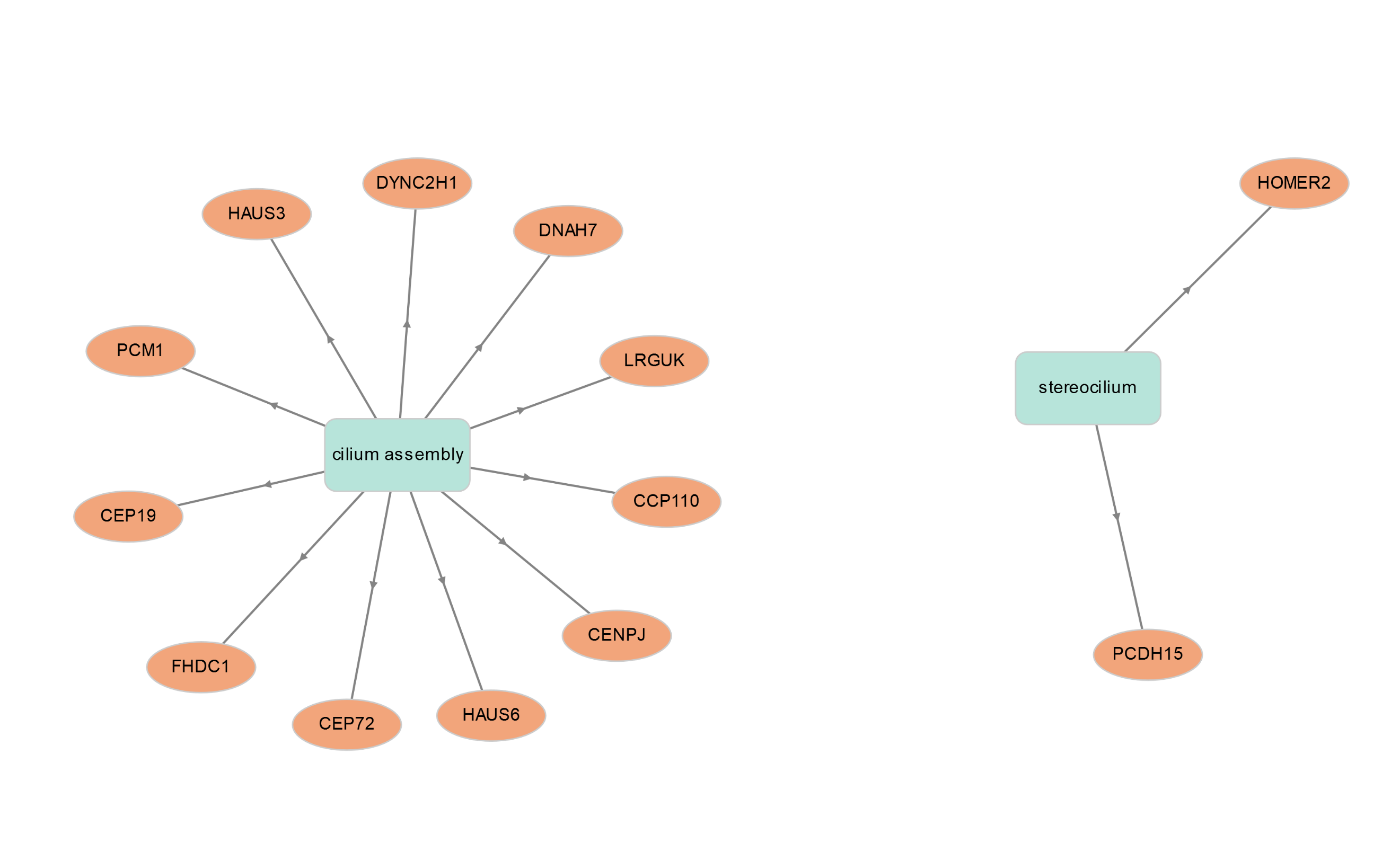
**

**Figure S3b: Gene ontology annotation of clinically significant GOterms in Sumatran tigers. Deleterious variants were observed in 44 genes included in custom list of GO terms relevant to known heritable conditions, immune and reproductive function. These genes were annotated for terms relevant to cilium structure. Network interaction graph produced by GOnet (**[**https://tools.dice-database.org/GOnet/**](https://tools.dice-database.org/GOnet/)**)**

**Table S9: Samples downloaded from Sequence Read Archive (SRA) comprised six cheetahs, one snow leopard and seven Sumatran tigers.**

| **Species** | **Sample** | **SRA ID** | **Sex** | **Population** | **Sequencing platform** | **Reference** |
| --- | --- | --- | --- | --- | --- | --- |
| Cheetah | CHEETAH_TZA3 | [SRR2737545](https://trace.ncbi.nlm.nih.gov/Traces/sra/?run=SRR2737545) | F | Tanzania | Illumina HiSeq 2000 | Dobrynin et al. 2015 |
| Cheetah | CHEETAH_TZA2 | [SRR2737544](https://trace.ncbi.nlm.nih.gov/Traces/sra/?run=SRR2737544) | M | Tanzania | Illumina HiSeq 2000 | Dobrynin et al. 2015 |
| Cheetah | CHEETAH_TZA1 | [SRR2737543](https://trace.ncbi.nlm.nih.gov/Traces/sra/?run=SRR2737543) | M | Tanzania | Illumina HiSeq 2000 | Dobrynin et al. 2015 |
| Cheetah | CHEETAH_NAM3 | [SRR2737542](https://trace.ncbi.nlm.nih.gov/Traces/sra/?run=SRR2737542) | M | Namibia | Illumina HiSeq 2000 | Dobrynin et al. 2015 |
| Cheetah | CHEETAH_NAM2 | [SRR2737541](https://trace.ncbi.nlm.nih.gov/Traces/sra/?run=SRR2737541) | M | Namibia | Illumina HiSeq 2000 | Dobrynin et al. 2015 |
| Cheetah | CHEETAH_NAM1 | [SRR2737540](https://trace.ncbi.nlm.nih.gov/Traces/sra/?run=SRR2737540) | F | Namibia | Illumina HiSeq 2000 | Dobrynin et al. 2015 |
| Snow leopard | SNOW | [SRR836372](https://trace.ncbi.nlm.nih.gov/Traces/sra/?run=SRR836372) | F | Captive, Korea | Illumina HiSeq 2000 | Cho et al. 2013 |
| Sumatran tiger | SUM_IND1 | [SRR7152379](https://trace.ncbi.nlm.nih.gov/Traces/sra/?run=SRR7152379) | M | Taman Safari, Indonesia | Illumina HiSeq 2500 | Liu et al. 2018 |
| Sumatran tiger | SUM_USA1 | [SRR7152382](https://trace.ncbi.nlm.nih.gov/Traces/sra/?run=SRR7152382) | F | Phoenix Zoo, USA | Illumina HiSeq 2500 | Liu et al. 2018 |
| Sumatran tiger | SUM_IND2 | [SRR7152383](https://trace.ncbi.nlm.nih.gov/Traces/sra/?run=SRR7152383) | F | Taman Safari, Indonesia | Illumina HiSeq 2500 | Liu et al. 2018 |
| Sumatran tiger | SUM_IND3 | [SRR7152384](https://trace.ncbi.nlm.nih.gov/Traces/sra/?run=SRR7152384) | F | Taman Safari, Indonesia | Illumina HiSeq 2500 | Liu et al. 2018 |
| Sumatran tiger | SUM_IND4 | [SRR7152385](https://trace.ncbi.nlm.nih.gov/Traces/sra/?run=SRR7152385) | M | Taman Safari, Indonesia | Illumina HiSeq 2500 | Liu et al. 2018 |
| Sumatran tiger | SUM_IND5 | [SRR7152386](https://trace.ncbi.nlm.nih.gov/Traces/sra/?run=SRR7152386) | F | Taman Safari, Indonesia | Illumina HiSeq 2500 | Liu et al. 2018 |
| Sumatran tiger | SUM_USA2 | [SRR7152388](https://trace.ncbi.nlm.nih.gov/Traces/sra/?run=SRR7152388) | M | Atlanta Zoo, USA | Illumina HiSeq 2500 | Liu et al. 2018 |

**Table S10: Genes associated with size in domestic species identified from a literature search**

| **Gene symbol** | **Gene name** | **First cited in** |
| --- | --- | --- |
| *ADAMTSL9-AS9* | ADAM metallopeptidase with thrombospondin type 1 motif 9 | Plassais *et al.*, 2019 |
| *ACSL4* | Acyl-CoA Synthetase Long Chain Family Member 4 | Plassais *et al.*, 2017 |
| *GHR1* | Growth Hormone Receptor 1 | Rimbault *et al.*, 2013 |
| *GHR2* | Growth Hormone Receptor 2 | Rimbault *et al.*, 2013 |
| *HNF4G* | Hepatocyte nuclear factor 4 gamma | Plassais *et al.*, 2017 |
| *HMAG2* | High-mobility group AT-hook 2 | Rimbault *et al.*, 2013 |
| *IGSF1* | Immunoglobulin superfamily, member 1 | Rimbault *et al.*, 2013 |
| *IGF1* | Insulin Growth Factor 1 | Sutter *et al.*, 2007 |
| *IGFBP2* | Insulin Like Growth Factor 2 MRNA Binding Protein 2 | Jones *et al.*, 2008 |
| *IGF1R* | Insulin-like growth factor-1 receptor | Hoopes *et al.*, 2012 |
| *IRS4* | Insulin Receptor Substrate 4 | Plassais *et al.*, 2017 |
| *LCORL* | Ligand dependent nuclear receptor corepressor | Plassais *et al.*, 2019 |
| *R3HDM1* | R3H domain containing 1 | Plassais *et al.*, 2019 |
| *SMAD2* | SMAD Family Member 2 | Rimbault *et al.*, 2013 |
| *STC2* | Stanniocalcin 2 | Rimbault *et al.*, 2013 |
| *ZNF608* | Zinc finger Protein 608 | Plassais *et al.*, 2019 |

**Table S11: Selected GO enrichment terms used to classify deleterious variants potentially implicated in reproductive success and overall health of captive bred big cats.**

**Table S12: Known heritable conditions affecting captive bred big cat species and relevant GOterms**
